# Organophosphate and Pyrethroid Hydrolase Activities of Mutant Esterases from the Cotton Bollworm *Helicoverpa armigera*

**DOI:** 10.1371/journal.pone.0077685

**Published:** 2013-10-29

**Authors:** Yongqiang Li, Claire A. Farnsworth, Chris W. Coppin, Mark G. Teese, Jian-Wei Liu, Colin Scott, Xing Zhang, Robyn J. Russell, John G. Oakeshott

**Affiliations:** 1 Research and Development Centre of Biorational Pesticides, College of Plant Protection, Northwest A&F University, Yangling, People’s Republic of China; 2 CSIRO Ecosystem Sciences, Canberra, ACT, Australia; 3 School of Biological Sciences, Australian National University, Canberra, ACT, Australia; 4 Cotton Catchment Communities CRC, Narrabri, NSW, Australia; University of Crete, Greece

## Abstract

Two mutations have been found in five closely related insect esterases (from four higher Diptera and a hymenopteran) which each confer organophosphate (OP) hydrolase activity on the enzyme and OP resistance on the insect. One mutation converts a Glycine to an Aspartate, and the other converts a Tryptophan to a Leucine in the enzymes’ active site. One of the dipteran enzymes with the Leucine mutation also shows enhanced activity against pyrethroids. Introduction of the two mutations *in vitro* into eight esterases from six other widely separated insect groups has also been reported to increase substantially the OP hydrolase activity of most of them. These data suggest that the two mutations could contribute to OP, and possibly pyrethroid, resistance in a variety of insects. We therefore introduced them *in vitro* into eight *Helicoverpa armigera* esterases from a clade that has already been implicated in OP and pyrethroid resistance. We found that they do not generally enhance either OP or pyrethroid hydrolysis in these esterases but the Aspartate mutation did increase OP hydrolysis in one enzyme by about 14 fold and the Leucine mutation caused a 4–6 fold increase in activity (more in one case) of another three against some of the most insecticidal isomers of fenvalerate and cypermethrin. The Aspartate enzyme and one of the Leucine enzymes occur in regions of the *H. armigera* esterase isozyme profile that have been previously implicated in OP and pyrethroid resistance, respectively.

## Introduction

There has been much debate in recent years over the genetic options available to insect pests to evolve resistance to chemical insecticides [1]–[3]. While large numbers of species have evolved such resistances, the biochemical and molecular mechanisms by which they do so appear to be quite limited. Very similar mutations conferring insensitivity on the molecular targets for particular classes of insecticide have been found across a wide range of species and resistance due to enhanced metabolism has almost always been found to result from elevated expression of detoxifying enzymes such as cytochrome P450s, esterases and glutathione S-transferases. Perhaps the most radical resistance mechanism has involved structural mutations in carboxylesterases which convert them to organophosphate (OP) hydrolases [4], [5] and, once again, there is evidence that the same mutations confer OP resistance on a few different species [6], [7], with recent reports suggesting that they could have the same effect in several others [8], [9]. This paper further explores the generality of the effect in conferring OP hydrolase activity on carboxylesterase enzymes and OP resistance on the host insects.

The two mutations in question have now been found to confer OP hydrolase activity and resistance in four species of higher Diptera (the blowflies *Lucilia cuprina* and *Lucilia sericata*, the house fly *Musca domestica* and the screwworm *Cochliomia hominivorax*) and one hymenopteran (the parasitic wasp *Anisopteromalus calandrae*) [4]–[7], [10]–[12]. While the efficiency of the mutant enzymes as OP hydrolases is low in kinetic terms it is nevertheless sufficient to confer major-gene OP resistance on individuals carrying either of them. Although both mutations protect against a wide range of OP insecticides, one, Gly137Asp in the *L. cuprina* E3 enzyme in which it has been most thoroughly characterised, gives stronger OP hydrolase activity and resistance than the other, Trp251Leu in *L. cuprina* E3, at least for the majority of (diethyl) OPs used commercially [13], [14].

Work on the *L. cuprina* enzyme shows that the Gly137Asp mutation compromises the native carboxylesterase activity of the enzyme [13], [14] and can cause fitness costs in the absence of OP insecticides [15]. Futhermore, this amino acid difference has not been reported as a naturally occurring substitution in any esterase sequences outside those implicated in OP resistance [8], [16], [17]. On the other hand, the Trp251Leu mutation has less effect on carboxylesterase activity, no fitness cost in the absence of OP insecticides has been reported for it, and indeed it existed at appreciable frequencies in *L. cuprina* even before OP insecticides were first used [18]. Genomic analyses also show that the Trp251Leu mutation occurs widely among insect esterase sequences whether or not the host species has been treated with OPs [8], [16], [17]. Intriguingly, in *L. cuprina* at least, this mutation also enhances the hydrolytic activity of E3 for the insecticidal isomers of several synthetic pyrethroid (SP) insecticides (which are also carboxylesters) [19], [20], although this is not in itself sufficient to confer SP resistance on this species [21].

The structure of the *L. cuprina* E3 enzyme provides a mechanistic explanation for the various activities of the wild-type and mutant versions of it [4], [5], [13], [22]. The Gly137 residue occurs in the oxyanion hole of the wild-type enzyme, where it stabilises the acyl-enzyme intermediate formed during carboxylester hydrolysis. The Gly137Asp mutation disrupts the normal function of the oxyanion hole, and hence the normal carboxylesterase activity of the enzyme, but it allows a water molecule to be oriented for nucleophilic attack on, and dephosphorylation of the phosphoryl-enzyme intermediate formed in the presence of OPs. The Trp251 residue sits in the acyl pocket of the wild-type enzyme and its substitution with Leu in the second mutant provides the additional space required to accommodate the trigonal bipyramidal structure of the phosphoryl intermediate (as opposed to the tetrahedral structure of the acyl intermediate). This mutation is less disruptive to the mechanism of carboxylester hydrolysis than the oxyanion hole change, and the effect depends on the geometry of the carboxylester substrate – hence its isomer-specific effect on SP hydrolysis.

Cui et al. [8], [9] transferred the two mutations to eight cloned esterases from other insect species in order to test whether they could potentially confer OP hydrolase activity more generally and, by extension, metabolic resistance on other species. There are in fact many cases where esterases have been implicated in metabolic resistance to OPs but for which the molecular basis is as yet unknown [23], [24]. Characterisation of the mutant esterases of Cui et al. [8], [9] expressed in a heterologous system showed that both mutations could indeed generate significant hydrolytic activity against certain OPs in several of the esterases. In fact some of the OP hydrolase activities obtained by Cui et al. [8], [9] were apparently higher than have been reported for *L. cuprina* E3. Notably also, the effects of the two mutations on carboxylesterase activity were quite variable and two cases were found where the Asp susbstitution in the presumptive oxyanion hole had little or no adverse effect on carboxylesterase activity.

In this study we have tested whether the two mutations could confer OP and SP hydrolase activity on eight esterases from the cotton bollworm *Helicoverpa armigera*. *H. armigera* is a major pest of cotton and other broad-acre and horticultural crops in Africa, Asia and Australia and metabolic resistances to OPs and SPs have arisen in many populations of the species ([25]–[27] and references therein). Esterases have been implicated in both these resistances although the specifics of the esterase genes involved and their molecular mechanisms have not yet been elucidated [3], [23], [28]. The eight esterases we have focused on are all from one particular clade of the carboxyl/cholinesterase family which several lines of evidence have suggested is involved in detoxifying functions, and possibly also OP and SP resistance [29]–[32].

## Materials and Methods

Clones of the eight wild-type esterase genes were described in Teese et al. [29] (and see also Wu et al. [30]). These cover all but two of the ten esterase genes so far attributed to Clade 1 of those authors (*CCE-001b*, *001c*, *001d*, *001f*, *001g*, *001h*, *001i* and *001j*, with *001a* proving difficult to express in active form and the *001e* clone lacking an intact C terminus; Teese et al. [32]). Mutations equivalent to Gly137Asp were introduced into all eight of these using the QuikChange Site-Directed Mutagenesis kit (Stratagene, USA). Mutations equivalent to Trp251Leu in E3 were also introduced into both the wild-type and Gly-Asp versions of five of these esterase genes by similar procedures *(001c*, *001g* and *001h* already containing the equivalent of Leu251; [29], [32]). Primers for the mutagenesis are given in Figure S1 and an alignment of the *H. armigera* esterases with E3 over the regions of the mutations is given in Figure S2. Notably, the residue at the 251 site is less conserved than that at the 137 site among the *H. armigera* esterases (as is also the case among other insect esterases [17], [29]), and a substitution of Leu for the Phe or Ileu commonly found in the 251-equivalent site of the *H. armigera* esterases may be less physicochemically radical than the same substitution into the Trp at this position in E3.

The various mutant genes were then transferred to baculovirus vectors and heterologously expressed in SF9 cells as per previously published methods [32]. The expressed enzymes were characterised for their phenotypes after native PAGE and staining for 1-naphthyl acetate hydrolytic activities as per the methods of Teese et al. [29] and the kinetics of their carboxylesterase activities with 1-naphthyl acetate as substrate were measured according to the methods of Teese et al. [32]. They were also tested for their hydrolytic activities against the model OPs dimethyl 4-methylumbelliferyl phosphate (dMUP) [13] and diethyl 4-methylumbelliferyl phosphate (dEUP, Sigma) by the fluorometric methods of Coppin et al. [17] and Teese et al. [32]. The dEUP assays also generated estimates of the titres of the enzymes, as explained in Coppin et al. [17]. High pressure liquid chromatography (HPLC) was also used to assay the enzymes’ hydrolytic activities against the eight resolved isomers of cypermethrin and the insecticidal 2(*S*)−α(*S*) isomer of fenvalerate, following the methods of Teese et al. [32]. The results from all these assays were then compared with those collected contemporaneously for the corresponding wild-type enzymes and the E3 wild-type and mutant controls as reported in Teese et al. [32].

## Results

**Figure 1:**
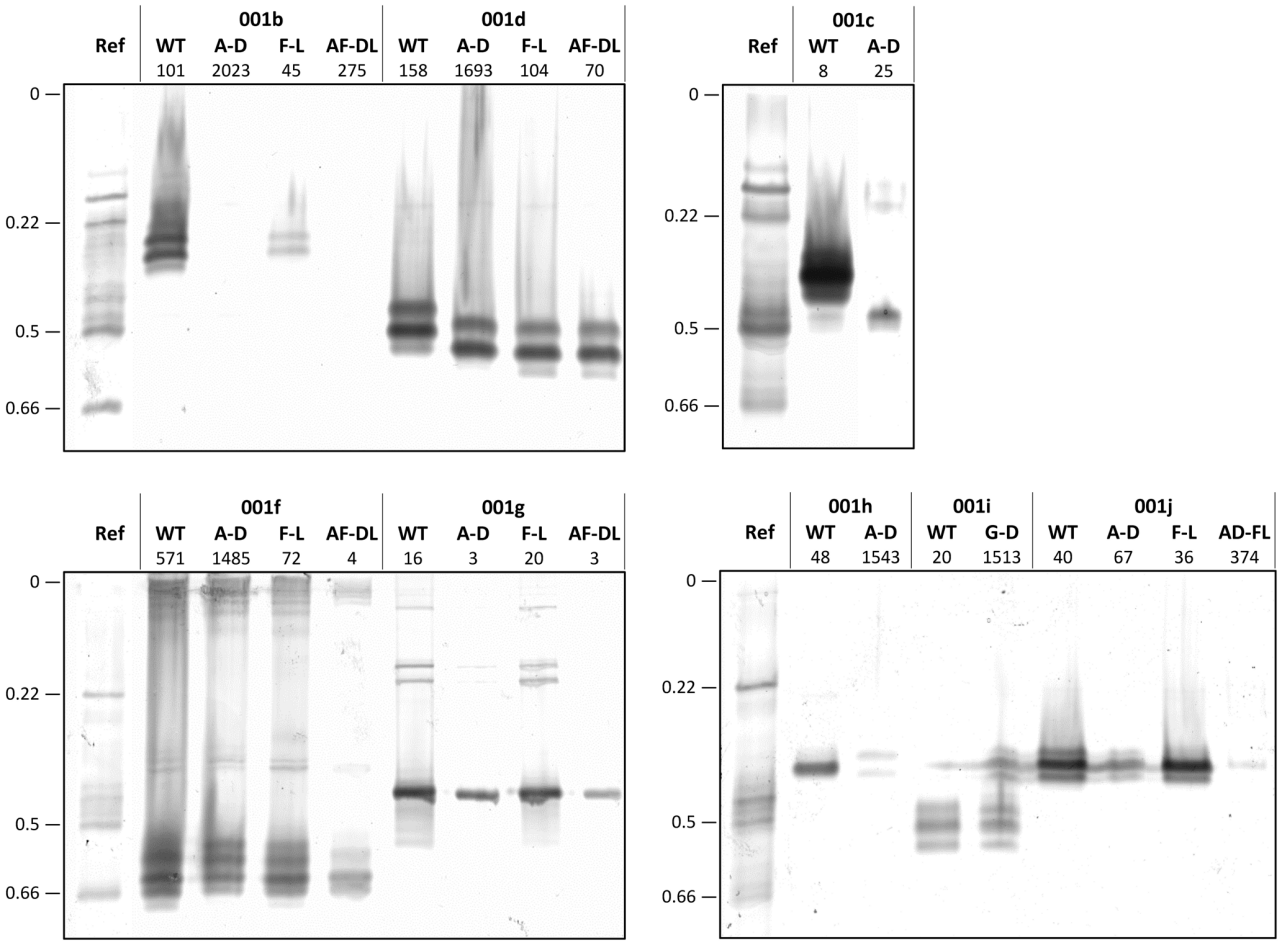
Native PAGE of the wild-type and mutant forms of the eight Clade 1esterases. Ref is a bulk homogenate of fourth instar larvae from the GR strain [13] and R_m_ values for some key Ref bands are given on the side of each panel. Amounts of enzyme loaded (number of active sites × 10^−11^ calculated from the titration values in Table S1) are given below the enzyme names; these amounts were varied across the enzymes in order to produce qualitatively similar staining intensities for as many enzymes as possible. Note that “10^−11^” was omitted from the description of enzyme amounts in the original presentation of the wild-type isozyme profiles in Figure 1 of Teese et al. [32].

Teese et al. [32] have previously shown that the wild-type versions of the eight *H. armigera* Clade 1 esterases under study are all produced in the baculovirus system in catalytically active forms which are readily detectable by native PAGE and staining with 1-naphthyl acetate. We find that the Asp mutation substantially reduces the intensities of most of these isozymes, albeit it still leaves detectable activities in all of them except 001b (Figure 1). A drastic drop in spectrophotometrically determined specific activity against 1-naphthyl acetate is also found for seven of the Asp mutants, the exception being the 001g mutant, which retains essentially the same level of activity as the wild-type enzyme (Table 1). Kinetic analysis shows that the Asp generally increases *K*_m_, the major exception in this case being 001f, for which the loss of activity is mainly associated with a drop in *k_cat_* (Table 1). Thus the overall pattern across the eight mutants generally agrees with the expectation from the prior work with other enzymes that the Asp mutation would compromise carboxylesterase activity, but there are significant differences among some of the enzymes in the nature and severity of the effect. This suggests that the mutation may be located in at least roughly equivalent locations in the tertiary structures of several of the esterases analysed here as it is in E3. However the various exceptions also suggest significant differences in the detail of the oxyanion hole configuration in several of the enzymes.

**Table 1:** Carboxylesterase activities for the wild-type and mutant forms of the eight *H. armigera* esterases with 1-naphthyl acetate as substrate.

Enzyme		Specificactivity		*k_cat_*		*K_m_*	
001b	Wt	47	(2)	63	(4)	199	(25)
	A125D	3	(0)	18	(4)	2056	(553)
	F236L	37	(1)	45	(2)	147	(13)
	AF/DL	2		na		na	
001c	Wt	214	(47)	522	(145)	712	(210)
	A127D	5	(4)	na		na	
001d	Wt	551	(119)	744	(162)	142	(18)
	A124D	19	(1)	na		na	
	F235L	593	(23)	1184	(72)	489	(45)
	AF/DL	10		na		na	
001f	Wt	110	(7)	108	(4)	4	(1)
	A127D	6	(0)	6	(0)	5	(1)
	F238L	116	(10)	117	(5)	6	(2)
	AF/DL	40	(33)	43	(36)	31	(10)
001g	Wt	436	(30)	444	(27)	4	(1)
	A127D	421	(48)	494	(51)	61	(7)
	F238L	257	(13)	246	(7)	1	(0)
	AF/DL	227	(67)	288	(81)	127	(36)
001h	Wt	71	(4)	96	(3)	9	(2)
	A125D	3	(0)	4	(1)	279	(29)
001i	Wt	20	(4)	24	(4)	74	(20)
	G130D	2	(0)	6	(1)	902	(318)
001j	Wt	502	(27)	569	(32)	46	(5)
	A125D	17	(3)	29	(6)	353	(52)
	F236L	359	(8)	550	(27)	241	(24)
	AF/DL	1		na		na	
E3	Wt	1173	(59)	1399	(70)	88	(10)
	G137D	650	(97)	763	(82)	34	(7)
	W251L	1234	(83)	1562	(76)	115	(12)

Specific activities (at 500 µM substrate) (sec^−1^) and estimates of *K*_m_ (µM) and *k_cat_* (sec^−1^) are shown. Estimates are based on an average of six replicates and standard errors for these estimates are also given in brackets. Values for the wild-type and corresponding mutants of *L. cuprina* E3 are also shown as controls. na, value could not be calculated.

The five enzymes into which we introduced the Leu mutation (001b, -1d, -1f, -1g and -1i) are all clearly evident in the isozyme analysis (Figure 1) and showed qualitatively similar activities with 1-naphthyl acetate as did the corresponding wild-type enzymes in the spectrophotometric assays (Table 1), the biggest differences being ∼40% drops in the activities of the mutants in 001g and 001j. The kinetic analyses also showed qualitatively similar *K*_m_ and *k*_cat_ values for the Leu mutants and wild type enzymes (Table 1). The results for this mutation in the *H. armigera* enzymes thus mirror those found in the *L. cuprina* E3 enzyme and the other esterases tested by Cui et al. [8], [9], supporting the notion that they sit in roughly equivalent positions in the acyl pockets of their respective active sites.

The activities of three of the five Asp/Leu double mutants agreed well with the activities of the respective Asp single mutants (Figure 1, Table 1). In these cases (001b, 001d and 001j) the Asp change had produced drastic drops in esterase activity as a single mutation, and it also did so in the double mutation. In another case (001f) Asp had also caused a significant drop in activity as a single mutant but the double mutant showed a less marked drop, yielding activity that was roughly intermediate between those of the Asp and Leu single mutants. In the remaining case (001g) the single Asp mutant had produce little change from wild type, whereas the Leu mutation had dropped activity by ∼40%, and in this case the double mutant showed a similar drop to the Leu mutant.

Table 2 shows large differences among the *H. armigera* esterases in terms of the effects of the Asp and Leu mutations on hydrolytic activities against the two OPs tested. In only three of the enzymes were significantly enhanced activities recovered from either of the mutations; these were 001c where the Asp mutation increased dEUP activity about 14 fold, 001d where the Leu mutation increased activity for each OP by 2–3 fold, and 001f where the double mutation increased the two activities about 3 fold. In the other enzymes the Leu mutation alone had little effect while the Asp change was generally deleterious either as a single or a double mutation. We do not know whether the smaller increases seen for 001d and 001f would be biologically significant but the large increase observed for 001c with dEUP yields turnover rates about 2 fold higher than those seen in the corresponding E3 mutant (Table 2) [13], [14], [32]. The value for 001c is low in enzymological terms (of the order of 0.2 min^−1^; Table 2) but, as noted earlier, the lower value for the E3 mutant is still sufficient to confer OP resistance on *L. cuprina*.

**Table 2:** Estimates of *kcat* (min^−1^.10^3^) for the wild-type and mutant forms of the eight *H. armigera* esterases with the OPs dEUP and dMUP as substrates.

Enzyme		*k_cat_* (dEUP)		*k_cat_* (dMUP)	
001b	Wt	24.6	(2.9)	15.5	(0.6)
	A125D	10.8	(0.1)	0.0	(0.0)
	F236L	14.2	(0.2)	29.3	(3.2)
	AF/DL	4.1	(0.1)	5.2	(0.2)
001c	Wt	13.0	(2.5)	26.5	(2.7)
	A127D	178.3	(4.2)	33.2	(7.4)
001d	Wt	9.4	(1.6)	11.4	(1.2)
	A124D	0.4	(0.0)	2.1	(0.2)
	F235L	25.7	(2.6)	38.5	(3.5)
	AF/DL	10.4	(0.6)	14.6	(0.4)
001f	Wt	14.0	(2.0)	16.6	(3.2)
	A127D	4.9	(0.1)	3.9	(0.5)
	F238L	12.3	(1.3)	17.8	(0.8)
	AF/DL	37.7	(4.5)	45.4	
001g	Wt	21.9	(1.7)	27.9	(1.8)
	A127D	25.2	(1.3)	16.3	(8.3)
	F238L	16.4	(0.6)	22.1	(0.8)
	AF/DL	24.5	(3.0)	nd	
001h	Wt	19.3	(0.7)	27.2	(3.8)
	A125D	0.5	(0.1)	2.0	(0.1)
001i	Wt	19.0	(3.5)	15.5	(2.1)
	G130D	1.0	(0.1)	3.3	(0.3)
001j	Wt	13.3	(1.1)	29.7	(2.2)
	A125D	2.0	(0.1)	0.4	(0.4)
	F236L	16.8	(2.5)	33.0	(0.5)
	AF/DL	0.7	(0.1)	7.5	(0.2)
E3	Wt	8.8	(1.0)	13.3	(0.7)
	G137D	92.4	(3.7)	144.3	(6.8)
	W251L	27.5	(3.3)	182.9	(7.3)

Estimates are based on an average of five replicates and standard errors for these estimates are also given in brackets. Values for the wild-type enzymes and the three E3 controls are taken from Teese et al. [32]. nd, not determined.

Table 3 shows activities against the insecticidal 2(*S*)−α(*S*) isomer of fenvalerate for the five enzymes with the Leu mutation in the acyl pocket, plus the respective wild-type enzymes. Activities for all but two of the enzymes were in the 0–2 min^−1^ range (the wild-type E3 value also being 2±2 min^−1^), the two exceptions being the Leu mutations in 001b (9±3 min^−1^) and 001d (6±4 min^−1^), which showed 5–6 fold enhancements over the respective wild-types. These values are still somewhat less than seen for the E3 Leu mutant (Table 3 and Heidari et al. [19]) but more than a log higher than the activities seen for the two OPs above.

**Table 3:** Estimates of turn over numbers (min^−1^) for the wild-type (Wt) and Leu mutants of five of the esterases with esfenvalerate and the eight isomers of cypermethrin.

		Esfenvalerate		Cypermethrin isomers															
Enzyme		2(*S*)-α(*S*)		1(*S*)*trans*-α(*S*)		1(*S*)*trans*-α(*R*)		1(*R*)*trans*-α(*S*)		1(*R*)*trans*-α(*R*)		1(*S*)*cis*-α(*S*)		1(*S*)*cis*-α(*R*)		1(*R*)*cis*-α(*S*)		1(*R*)*cis*-α(*R*)	
001b	Wt	1.5	(1.5)	37.3	(1.6)	32.3	(2.0)	4.4	(1.7)	7.9	(0.5)	2.1	(0.3)	5.8	(3.6)	3.9	(3.9)	5.4	(1.5)
001b	F236L	9.1	(3.3)	49.1	(6.9)	24.1	(2.4)	6.3	(1.2)	3.8	(3.5)	0.9	(0.7)	7.7	(3.5)	5.7	(4.5)	9.7	(3.2)
001d	Wt	0.8	(0.5)	16.0	(3.6)	24.6	(4.4)	6.2	(3.7)	3.2	(0.9)	10.1	(9.9)	1.9	(0.9)	4.1	(2.7)	3.4	(1.4)
001d	F235L	5.5	(3.8)	53.8	(11.3)	55.9	(12.9)	1.7	(1.7)	7.1	(1.9)	0.0	(0.0)	11.1	(2.1)	18.3	(3.3)	14.0	(3.6)
001f	Wt	0.0	(0.0)	3.0	(0.2)	2.8	(0.2)	0.0	(0.0)	0.4	(0.0)	0.2	(0.2)	0.9	(0.9)	1.3	(1.3)	2.7	(0.9)
001f	F238L	0.4	(0.4)	1.9	(1.2)	0.8	(0.4)	1.8	(1.2)	2.3	(1.1)	0.0	(0.0)	4.3	(1.7)	5.7	(0.9)	6.2	(0.1)
001g	Wt	17.0	(11.4)	12.9	(6.7)	12.8	(2.2)	0.0	(0.0)	8.8	(8.8)	1.3	(1.3)	15.7	(0.3)	9.3	(9.3)	23.2	(12.3)
001g	F238L	1.8	(1.8)	2.0	(0.8)	8.8	(4.9)	1.6	(1.6)	12.6	(3.2)	1.4	(1.4)	8.2	(8.2)	4.9	(0.9)	9.1	(9.1)
001j	Wt	1.8	(1.8)	32.9	(2.9)	55.1	(3.9)	0.5	(0.5)	9.1	(3.6)	2.2	(1.1)	9.0	(3.5)	11.6	(3.0)	12.4	(1.9)
001j	F236L	0.0	(0.0)	38.5	(7.1)	79.6	(9.2)	0.4	(0.4)	13.6	(4.0)	2.1	(2.1)	8.0	(5.2)	9.1	(6.0)	13.1	(0.9)
E3	Wt	1.9	(1.9)	22.3	(2.5)	11.5	(5.9)	1.8	(1.8)	5.7	(5.7)	0.0	(0.0)	14.8	(1.8)	18.5	(18.5)	10.6	(10.6)
	W251L	61.4	(26.6)	108.8	(14.5)	28.3	(4.2)	7.5	(5.4)	11.7	(6.6)	4.1	(2.3)	12.1	(7.7)	35.5	(5.8)	20.0	(8.0)

Estimates are based on an average of three replicates and standard errors for these estimates are also given in brackets. Values for the wild-type enzymes and two E3 controls are taken from Teese et al. [32].

Table 3 also gives the corresponding data for the eight isomers of cypermethrin. As with fenvalerate, there was no general trend for the Leu mutation to increase activities against the cypermethrin isomers, although there were several isomer-specific increases in particular enzymes. Values for the two most insecticidal isomers, 1*(R)trans−α(S)* and 1*(R)cis−α(S)*, were relatively low (up to 18±3 min^−1^, but generally considerably less), but the Leu mutation significantly improved activity for the former in 001f (wild-type levels being undetectable) and for the latter in 001d and 001f (both ∼4 fold). As for fenvalerate, these improvements were lower than those for E3 Leu but activities were still ∼2 logs higher than the OP values above.

## Discussion

There is a significant discrepancy between our data and those of Cui et al. [8], [9] in respect of the OP hydrolase activities of the Asp and Leu mutants. They tested eight enzymes from five insect orders, including two other lepidopteran esterases (from *Bombyx mori* and *Spodoptera litura*) from the same clade (Clade 1) as the eight studied here. Asp and Leu mutations in most of their enzymes, including both the lepidopteran esterases, significantly increased OP hydrolase activity. In our case each mutation alone only increased activity in one enzyme (001c and 001d), with the combination also increasing activity in another one (001f). Their increases were also quite large; most of their wild-type enzymes had activities below their level of detection whereas many of their mutants had activities that were more than a hundred fold higher than that of the corresponding E3 mutant. Our increases are much smaller, the highest activity among our mutants being just about 2 fold higher than that of the equivalent E3 enzyme. Their results suggest that the Asp mutation in particular could be a very common mechanism for metabolic resistance to OPs in insects. Our data suggest such a mechanism may have much less generality.

We do not know why there should be such a difference between the two studies and it is possible that our *H. armigera* enzymes are unusual in not showing such a dramatic effect. As noted earlier, some of the wild-type *H. armigera* esterases already had a Phenylalanine or Isoleucine residue at the 251 equivalent position, so a substitution to Leucine may have a less dramatic effect than the Trp251Leu change in E3 and some of the enzymes made by Cui et al [8], [9]. However this could only account for some of the differences between studies in respect of the Leu substitution, and none in respect of the Asp substitution. Two methodological differences between the studies are therefore also worth noting in this context. Firstly we note that Cui et al. [8], [9] expressed their enzymes as Histidine-tagged fusions in *E. coli*, which we have found problematic for the proper folding of Clade 1 and most other insect esterases (JWL, CAF and JGO unpublished data and see also below), whereas our enzymes were untagged and baculovirus expressed, which should produce properly folded protein. They also determined enzyme concentrations from total protein assays on their purified enzymes, which would not distinguish properly folded from inactive enzyme forms, whereas our titrations are specific for properly folded molecules [13]. At the least, we suggest that our results show the two mutations to be less generally beneficial for OP hydrolase activity than are suggested by Cui et al. [8], [9].

Clearly structural analysis of the *H. armigera* esterases could help elucidate these issues. Unfortunately empirical structures are only currently available for a few insect esterases (acetylcholinesterases from *Drosophila* and some other insects, juvenile hormone esterase from *Manduca sexta* and E3 [22], [23], [33], [34]), none of which show >30% amino acid identity to the *H. armigera* Clade 1 esterases. Homology modelling of the latter enzymes at the level of resolution required to understand the effects of individual mutations is therefore not yet possible. Instead we have tried to express three of the *H. armigera* esterases (001c, -1f and -1i) in *E. coli* in active form in amounts amenable to crystallography. We find that all three are mainly expressed as inactive aggregates under a range of genetic and growth conditions. We are currently using *in vitro* evolution technology as per Jackson et al [22] to develop an 001f variant which will fold sufficiently well for crystallographic analysis.

It is not clear whether the Asp version of *H. armigera* 001c, the enzyme showing the biggest (∼14 fold) improvement in OP hydrolase activity in our set, has any relevance to OP resistance in this species. On the one hand, 001c migrates in the same relative mobility zone (R_m_ 0.39–0.43) under native PAGE as esterase isozymes previously associated with OP resistance (whereas the 001d and 001f isozymes in which the mutations yielded much smaller improvements do not). On the other hand, the Asp mutation greatly reduces the 1-naphthyl acetate activity of 001c (Figure 1; Table 1), which contrasts with the more intense staining of this isozyme zone in OP resistant compared to susceptible larvae [31]. However the latter difference does not preclude a role for 001c Asp in resistance, since native western blotting showed that there was more Clade 1 enzyme present in this zone in resistant material, and several other Clade 1 and other esterases also migrate to the same zone [29]–[32]. Sequencing of *001c* genes from OP susceptible and resistant strains will be required to resolve this issue.

Only one of the three isozymes in which the Leu mutation was found to increase activity against the most insecticidal isomers of fenvalerate or cypermethrin (001d but not 001b or 001f) migrates to an isozyme zone (R_m_ 0.46–0.51) previously associated with pyrethroid resistance [31]. This zone was also more intensely staining in SP resistant than susceptible material [31]. In this case our data are entirely consistent with these electrophoretic and western phenotypes, since the Leu mutation had no adverse effect on 1-naphthyl acetate activity (Figure 1; Table 1). Sequencing of *001d* genes in SP susceptible and resistant strains would also now seem worthwhile.

## Supporting Information

Figure S1 **PCR primers used to generate the mutations.** Target codons are underlined, with mutated nucleotides given in lower case.
(TIFF)

Figure S2 **Cropped alignment of Clade 1** ***H. armigera*** **esterases and** ***L. cuprina*** **E3 (Accession No U56636).** Residues aligning with E3 G137 and W251 are shaded grey and red respectively. The alignment was made using ClustalW but an equivalent alignment of the shaded residues was also found using Muscle.
(TIF)

Table S1 Estimates of the (µM) concentration of active sites for the wild type and mutant forms of the eight *H. armigera* esterases and the three E3 controls using the dEUP titration method of Coppin et al. [17]. Estimates are based on an average of three replicates and standard errors for these estimates are also given. Values for the wild-type *H. armigera* enzymes and the three E3 controls are taken from Teese et al. [32].
(DOCX)
