# Supplementary figures and images for: Organophosphate and Pyrethroid Hydrolase Activities of Mutant Esterases from the Cotton Bollworm Helicoverpa armigera
Source: PLoS One. 2013 Oct 29;8(10):e77685. doi: 10.1371/journal.pone.0077685 (PMC3812244; doi:10.1371/journal.pone.0077685)

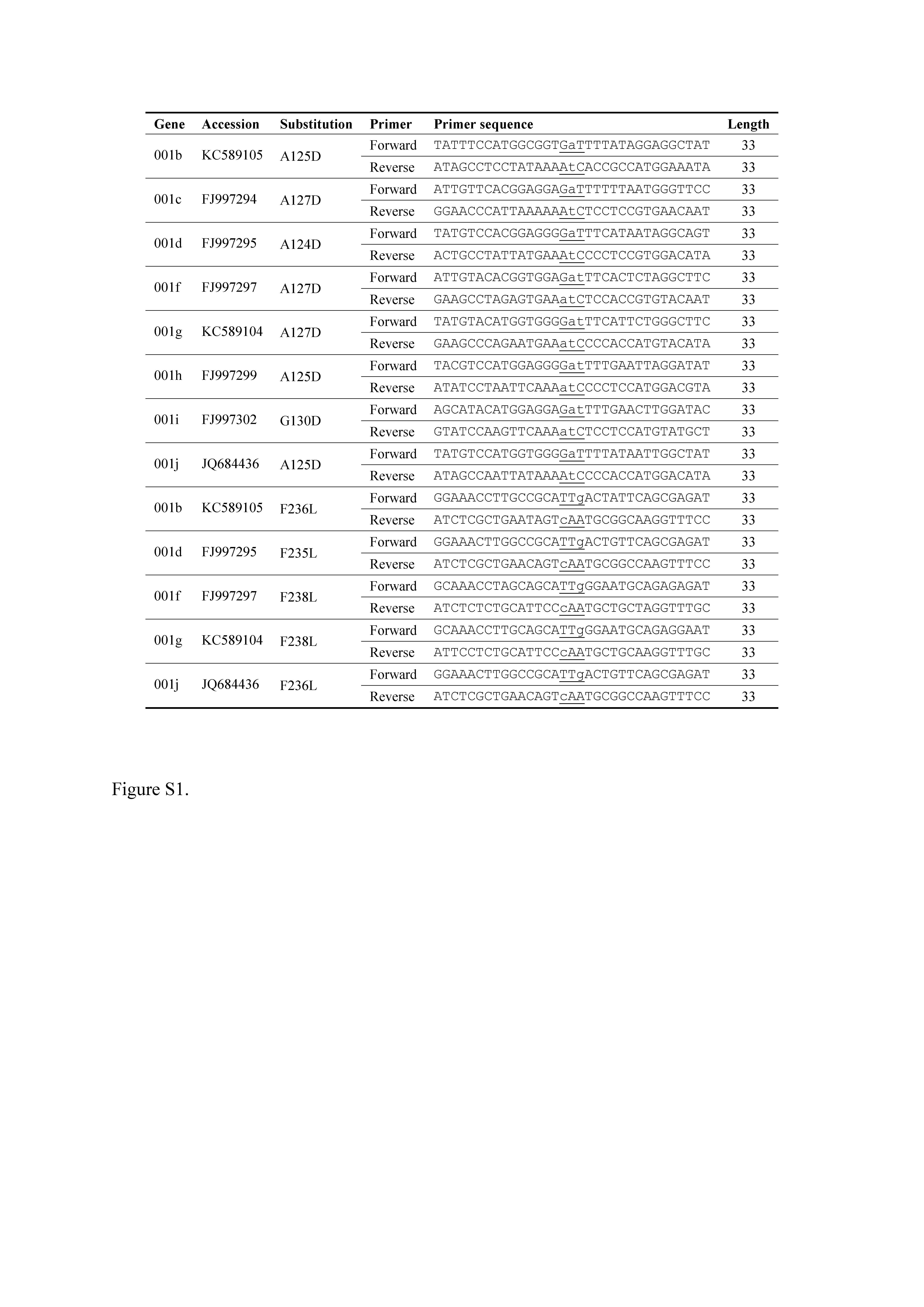

Supplement: Figure S1 — PCR primers used to generate the mutations. Target codons are underlined, with mutated nucleotides given in lower case. (TIFF) [file pone.0077685.s001.tiff]
