# Supplementary material for: Organophosphate and Pyrethroid Hydrolase Activities of Mutant Esterases from the Cotton Bollworm Helicoverpa armigera
Source: PLoS One. 2013 Oct 29;8(10):e77685. doi: 10.1371/journal.pone.0077685 (PMC3812244; doi:10.1371/journal.pone.0077685)
Supplement: Table S1 — Estimates of the (µM) concentration of active sites for the wild type and mutant forms of the eight H. armigera esterases and the three E3 controls using the dEUP titration method of Coppin et al. [17]. Estimates are based on an average of three replicates and standard errors for these estimates are also given. Values for the wild-type H. armigera enzymes and the three E3 controls are taken from Teese et al. [32]. (DOCX) [file pone.0077685.s003.docx]

**Table S1.** Estimates of the (µM) concentration of active sites for the wild type and mutant forms of the eight *H. armigera* esterases and the three E3 controls using the dEUP titration method of Coppin et al. [[17](#_ENREF_17)]. Estimates are based on an average of three replicates and standard errors for these estimates are also given. Values for the wild-type *H. armigera* enzymes and the three E3 controls are taken from Teese et al. [[32](#_ENREF_32)].

|  | **Enzyme** | | **Titre** | |
| --- | --- | --- | --- | --- |
|  | 001b | Wt | 3.7 | (0.1) |
|  | 001b | A125D | 16.0 | (0.3) |
|  | 001b | F236L | 2.5 | (0.0) |
|  | 001b | AF/DL | 5.7 | (0.3) |
|  | 001c | Wt | 6.1 | (1.2) |
|  | 001c | A127D | 1.0 | (0.7) |
|  | 001d | Wt | 5.1 | (1.1) |
|  | 001d | A124D | 14.8 | (0.4) |
|  | 001d | F235L | 1.4 | (0.0) |
|  | 001d | AF/DL | 2.9 | (0.5) |
|  | 001f | Wt | 9.6 | (0.3) |
|  | 001f | A127D | 13.7 | (0.4) |
|  | 001f | F238L | 3.0 | (0.1) |
|  | 001f | AF/DL | 1.3 | (1.1) |
|  | 001g | Wt | 0.9 | (0.1) |
|  | 001g | A127D | 0.2 | (0.0) |
|  | 001g | F238L | 1.7 | (0.0) |
|  | 001g | AF/DL | 0.3 | (0.1) |
|  | 001h | Wt | 2.4 | (0.0) |
|  | 001h | A125D | 12.2 | (1.6) |
|  | 001i | Wt | 1.9 | (0.3) |
|  | 001i | G130D | 15.7 | (0.9) |
|  | 001j | Wt | 2.2 | (0.1) |
|  | 001j | A125D | 2.8 | (0.5) |
|  | 001j | F236L | 2.0 | (0.0) |
|  | 001j | AF/DL | 6.9 | (0.5) |
|  | E3 | Wt | 1.4 | (0.0) |
|  | E3 | G137D | 1.1 | (0.1) |
|  | E3 | W251L | 1.0 | (0.0) |
